# Effect of Curing Temperature on High-Strength Metakaolin-Based Geopolymer Composite (HMGC) with Quartz Powder and Steel Fibers

**DOI:** 10.3390/ma15113958

**Published:** 2022-06-02

**Authors:** Qiang Li, Shikun Chen, Yajun Zhang, Yunjin Hu, Quanlin Wang, Quan Zhou, Yongmao Yan, Yi Liu, Dongming Yan

**Affiliations:** 1 Key Laboratory for Technology in Rural Water Management of Zhejiang Province, Zhejiang University of Water Resources and Electric Power, Hangzhou 310018, China; liq@zjweu.edu.cn; 2 College of Civil Engineering and Architecture, Zhejiang University, Hangzhou 310058, China; dmyan@zju.edu.cn; 3 Key Laboratory of Rock Mechanics and Geohazards of Zhejiang Province, Shaoxing University, Shaoxing 312000, China; huyunjin@tsinghua.org.cn (Y.H.); quanlin8wang@163.com (Q.W.); 4 Zhejiang Jiaotou Shengxing Mining Co., Ltd., Shaoxing 312432, China; sxky@cncico.com; 5 Shanxi Sanjian Group Co., Ltd., Changzhi 046000, China; yanym@sxcig.com; 6 Institute for Composites Science Innovation, School of Materials Science and Engineering, Zhejiang University, Hangzhou 310027, China; liuyimse@zju.edu.cn

**Keywords:** geopolymer, metakaolin, curing temperature, high strength

## Abstract

Geopolymer is a new type of synthesized aluminosilicate material. Compared with ordinary Portland cement, it has better fire resistance and durability, and is more environmentally friendly. In this paper, a high-strength metakaolin-based geopolymer composite (HMGC) has been developed by utilizing quartz powder and steel fibers. The optimization compositions and effect of curing temperatures (from ambient temperature to 90 °C) on the strength performance of the HMGC is studied. The optimized 1-day compressive strength of the HMGC can reach 80 MPa, and the 3-day compressive strength is close to 100 MPa (97.49 MPa). Combined with XRD, FTIR, SEM and MIP characterization, the mechanisms behind the strength development under different curing temperatures are analyzed. The results show that heat curing can significantly speed up the process of geopolymerization and increase the early strength of the HMGC. However, long-term heat curing under high temperature (such as 90 °C, 7 days) would reduce the mechanical strength of the HMGC. Prolonged high-temperature curing increases the pores and micro-defects in the gel phase of the HMGC, which may be attributed to chemical shrinkage. Thus, the curing temperature should be carefully controlled to make a HMGC with better performance.

## 1. Introduction

With the development of modern society, higher standards are required for the performance of civil engineering materials. The massively-used concrete grade has changed from C30 in the last century to C50–C60 nowadays. At present, through special material design and curing methods, the strength of ordinary Portland cement (OPC) concrete can reach as high as 100 MPa and above. However, the production of OPC materials generates a large amount of CO_2_. So, geopolymer concrete and composites with high strength and low carbon emissions have become popular research topics.

Sathonsaowophak et al. [1] studied the fineness of bottom ash, the liquid alkaline/ash ratio, the sodium silicate/NaOH ratio and the NaOH concentration on the compressive strength of geopolymer mortar, and obtained the relatively high strength (58 MPa) of geopolymer mortar with the water/ash ratio of 0.03. Chindaprasirt et al. [2] changed the mass ratio of sodium silicate to NaOH and the concentration of NaOH, and obtained the high-calcium fly ash-based geopolymer mortar (the low sodium silicate to NaOH ratio) with a maximum compressive strength of 65 MPa. Guo et al. [3] found that a high compressive strength (about 70 MPa) was obtained when the C-type fly ash was activated with a mixed alkali activator at the appropriate molar ratio (1.5) of SiO_2_/Na_2_O and the high the mass proportion of Na_2_O to CFA. Zivica et al. [4] prepared fresh mixtures and the compressive strength of the metahalloysite-based geopolymer after 24 h was 76.2 MP by applying a compaction pressure of 300 MPa. Bagheri et al. [5] optimized four different independent factors including aggregate content, sodium hydroxide concentration, curing time and curing temperature and obtained the 69.3 ± 5.3 MPa and 76.2 ± 3.6 MPa at 2 and 7 days for the specimen with low GBFS content, high oven curing temperature and long oven curing time. Vafaei et al. [6] proposed a synthesis method of alkali-activated geopolymer based on waste glass powder, and finally obtained a maximum compressive strength (87 MPa) of geopolymer with high calcium aluminate cement content and appropriate Na_2_O content (10%). Duan et al. [7] found that adding silica fume can enhance the strength of geopolymer, and the compressive strength could reach 90 MPa when 30% fly ash was replaced by silica fume. Li Dunxing [8] studied the effect of mineral powder content on the properties and structure of metakaolin-based geopolymers, and obtained a high compressive strength (102.3 MPa) when 30% metakaolin was replace by slag for 28 days. Khan et al. [9] obtained a 28-day compressive strength of 108 MPa when 50% fly ash was replaced by slag. Atis et al. [10] selected the thermal curing temperature, thermal curing time and alkali (Na) concentration as the influencing parameters of the strength, and finally obtained a high compressive strength of about 120 MPa when the geopolymer contained 14% Na content at 115 °C heat curing and for 24 h duration. Xie Suisui [11] used water glass and sodium hydroxide as alkali activators and metakaolin as the raw material, and added 0.5 wt.% aluminum tripolyphosphate to improve the strength, and the strength was as high as 156 MPa.

Quartz powder and steel fibers have been widely used in the preparation of ultra-high-performance concrete [12,13,14,15,16]. The incorporation of steel fibers can greatly improve the ductility and flexural properties of concrete [17]. Quartz powder mainly plays the role of providing nucleation sites and optimizing particle packing in concrete [18]. Kathirvel et al. [19] used quartz powder and steel fibers to prepare ultra-high performance geopolymer concrete and showed that the compressive strength increased with the addition of quartz powder and steel fibers, which was mainly because the quartz powder improves the particle packing of concrete and the crack bridging effect of steel fibers. Rashad et al. [20] also showed that, in addition to providing a large amount of silica, quartz powder can fill the space of hardened paste, thereby reducing the pores and improving the compressive strength.

Curing conditions have the significant effect on geopolymer properties, microstructures and even geopolymerization [21,22,23]. Nath et al. [24] shown that the chemical reaction rate of geopolymers would be slow if heat curing was not used. Kaplan et al. [21] studied the properties of fly-ash-based geopolymers with quartz powder under different curing conditions, and the results showed that increasing the curing temperature and curing time can improve the geopolymerization and improve the mechanical strength of the geopolymer. Previous studies have demonstrated the excellent effect of quartz powder and steel fibers on the properties of geopolymers. However, there is no report on the effect of curing temperature on the properties of metakaolin based geopolymers with the coexistence of steel fibers and quartz powder. Therefore, corresponding research needs to be carried out to explore the effect of curing temperature on the HMGC.

The above studies show that high-strength geopolymer materials can be obtained by material design. Based on these works, this paper develops a new type of high-strength metakaolin-based geopolymer composite (HMGC) by utilizing quartz powder and steel fibers. The 1-day compressive strength can reach 80 MPa, and the 3-day compressive strength is close to 100 MPa (97.49 MPa). In this paper, the effect of curing temperature (from ambient temperature to 90 °C) on the compressive and flexural strength of the proposed HMGC is also studied. Combined with XRD, FTIR, SEM and MIP characterizations, the underlying mechanisms behind the effect of temperature are analyzed as well. 

## 2. Materials and Methods

### 2.1. Materials

Metakaolin is an industrial-grade product produced by BASF. The chemical composition of metakaolin was determined by X-ray fluorescence spectroscopy (XRF). The specific chemical composition of oxides is shown in Table 1. The content of SiO_2_ was 57.46%, and the content of Al_2_O_3_ was 39.81%. The average particle size of metakaolin was 5.89 μm and the 90% passing particle size was 13.60 μm, obtained by a laser particle size analyzer.

The alkali activator solution was mixed with sodium water glass solution and flake NaOH. The sodium water glass solution used was a commercially-available, industrial grade water glass. The content of SiO_2_ was 26%, the content of Na_2_O was 8.2% and the rest was water. The flake NaOH was produced by Sinopharm Chemical Reagent Co., Ltd. (Shanghai, China), and the purity was above 96%. The alkali activator solution used in this test was mixed, stirred and placed at a temperature of 20 ± 5 °C for 24 h before the making process.

**Figure 1:**
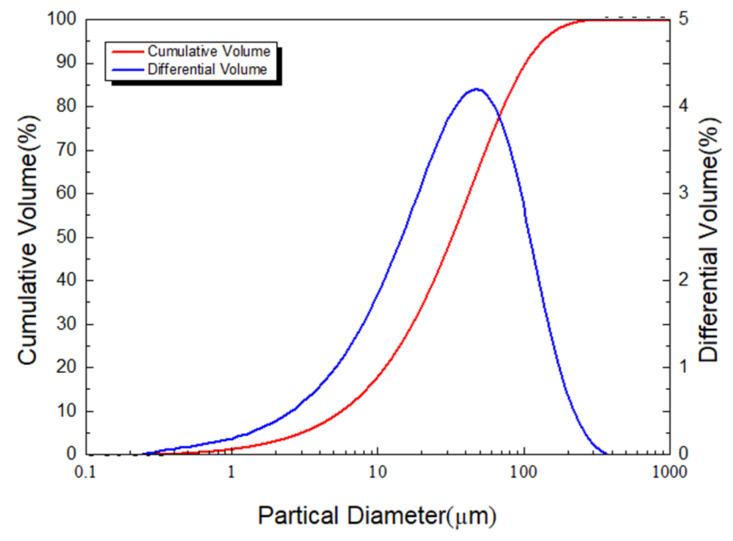
Quartz powder particle size distribution obtained by BT-9300ST laser particle size analyzer.

The quartz powder was produced by Gongyi Runjia Water Purification Material Sales Co., Ltd., (Gongyi, China). The specific surface area measured by the laser particle size analyzer was 203.8 m^2^/kg and the detailed particle size distribution is shown in Figure 1. 

The copper-coated steel fibers were produced by Ganzhou Daye Metallic Fibres Co., Ltd., (Ganzhou, China), and the physical parameters are shown in Table 2.

### 2.2. Mix Ratio and Preparation of HMGC

#### 2.2.1. Mix Ratio Design

The HMGC mix ratios refer to previous research results [25]. In order to determine the content of quartz powder and steel fibers, the strength and workability of geopolymers with quartz powder and steel fibers, respectively, were compared, as shown in Table 3 and Table 4. 

#### 2.2.2. Preparation of HMGC

Firstly, the metakaolin powder and alkali activator were mixed in the mixing pot for 2 min. Then, the quartz powder was quickly poured into the pot and blended for another 2 min. If steel fibers were added, they were poured into the mixing pot and mixed for 2 min. Finally, the mixture was cast into the mold and vibrated to eliminate air bubbles. The molds were sealed with plastic wrap to prevent water loss. For specimens used in composition optimization, the molds were cured in a curing room with a temperature of 20 ± 2 °C and a curing humidity of more than 90%. After curing for 24 h, the specimens were demolded and cured in the same environment for another 6 days before testing. For specimens used in the curing temperature study, the molds were firstly cured in the curing room as above for 5 h, and they were then demolded and cured at different temperatures. Three types of curing process were used. The first type consisted of the specimens being kept in the same curing room (20 °C). The second type consisted of the specimens being placed in a sealed box with humidity of more than 90% and cured in an oven with a temperature of 60 °C. The third type is similar to the second type, but differed in curing temperature (90 °C). Then, the HMGC specimens were cured until testing at 1 d, 3 d and 7 d.

### 2.3. Experiment Method

#### 2.3.1. Determination of HMGC Setting Time

To obtain a rational curing process under different temperatures, the setting time of the HMGC with optimized composition were measured by the standard Vicar instrument according to GB T1346-2011 [26]. The test molds with fresh mixtures were cured in a curing room (ambient temperature) or an oven (40 °C, 60 °C, 80 °C and 90 °C) between measurements.

#### 2.3.2. The Mechanical Properties of HMGC

The flexural strength and compressive strength of the HMGC were tested, referring to GB/T 17671-1999 [27]. In Section 3.1, the compressive strength was tested using the specimens of 40 × 40 × 40 mm^3^. In Section 3.2, Section 3.4 and Section 3.5, the flexural strength was tested using specimens of 40 × 40 × 160 mm^3^. The compressive strength was tested using broken specimens taken from the flexural test and tested with a loading surface of 40 × 40 mm^2^. Three specimens were tested, and the mean value and standard deviation of experimental results were presented.

#### 2.3.3. Phase Analysis—X-ray Diffraction Test (XRD)

The XRD test was performed using D8 Advance, with an emitter voltage of 40 kV, a tube current of 40 mA and a 2θ range of 5–80°. 

#### 2.3.4. Morphology Analysis—Scanning Electron Microscope Experiment

Scanning electron microscopy with a 3.0 kV voltage field was used to analyze the microscopic morphology of the HMGC. 

#### 2.3.5. Structural Gene Analysis—Infrared Spectroscopy (FTIR)

The Nicolet AVA TAR370-infrared spectrometer with a wavelength range of 4000–400 cm^−1^.

#### 2.3.6. Pore Structure Analysis—Mercury Intrusion Porosimetry (MIP)

The Auto Pore IV9510 automatic mercury porosimeter was used, in which the advanced contact angle was 130.0° and the mercury surface tension was 485.0 dynes/cm. 

## 3. Results

### 3.1. Optimization of Quartz Powder Content

**Figure 2:**
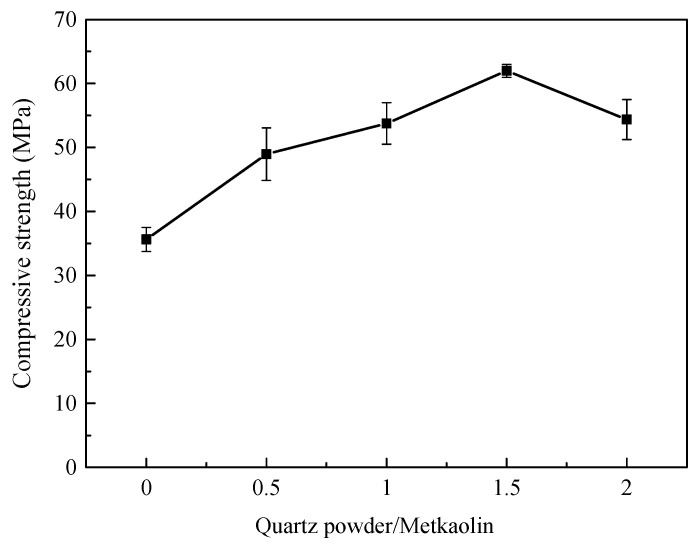
Compressive strength of HMGC with different quartz powder contents.

Figure 2 shows the compressive strength of the HMGC with different quartz powder contents after curing for 7 days. It can be seen from the figure that with the increase of quartz powder content, the compressive strength of HMGC first increases and then decreases. When the quartz powder/metakaolin ratio is 1.5, the compressive strength of HMGC reaches the maximum value of 61.98 MPa which is mainly because quartz powder can fill the space inside the skeleton of the hardened microstructure [20]. However, with the increase of quartz powder, the fluidity of the HMGC gradually decreases. When the ratio reaches 2.0, it becomes hard to mix. The bubbles were difficult to remove from the fresh mixture and more pores were formed in the specimens. The strength thus decreased. Therefore, the optimized quartz powder/metakaolin ratio is 1.5. 

### 3.2. Optimization of Steel Fiber Content

**Figure 3:**
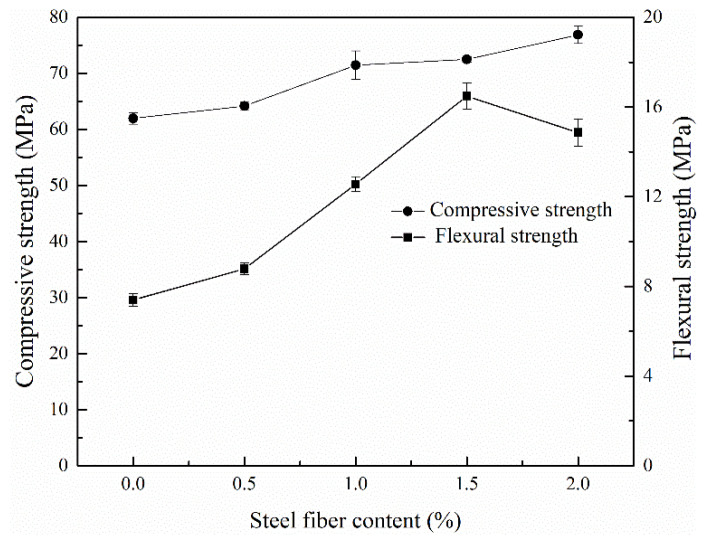
Compressive strength and flexural strength of HMGC with different steel fiber contents.

Figure 3 shows the variation of compressive and flexural strength of HMGCs with different steel fiber contents. It can be seen that the compressive strength of HMGCs gradually increases with the increase of fiber content. However, the flexural strength shows a trend of first increasing and then decreasing with the increase of fiber content. When the fiber content exceeds 1.5 vol.%, the flexural strength tends to decrease. For this composition, the fiber content was too high to be well dispersed, thus making the fibers agglomerate together, which lead to a decrease in flexural strength. Therefore, the optimized content of steel fibers is selected to be 1.5 vol.% for the later experiments of this paper.

### 3.3. Setting Time of HMGC

**Figure 4:**
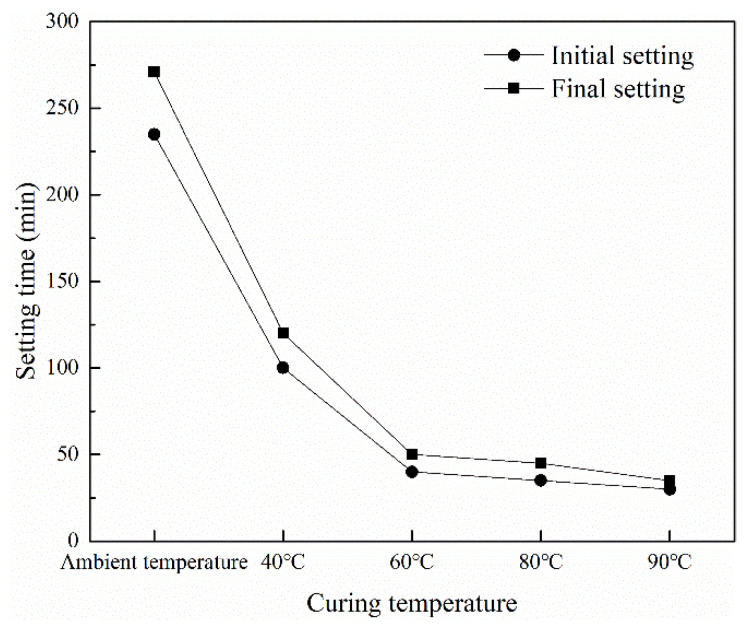
The setting time of HMGC at different temperatures.

Figure 4 shows the variation of the setting time of the HMGC at different temperatures. It can be seen that the setting time of the HMGC gradually decreases with the increase of temperature. At an ambient temperature, the initial setting time of the HMGC was 235 min, and the final setting time was 271 min. The setting time at room temperature shows that the HMGC has better early hardening properties. When the temperature was raised to 90 °C, the initial setting time of the HMGC was 30 min, and the final setting time was 35 min. The increase of temperature shortened the setting time of HMGC. This result could be attributed to the thermodynamic effect that the increase of temperature accelerates the chemical reaction rate of dissolution of powders and polycondensation processes, which can promote the formation of hard structures [21]. The proposed HMGC could harden in 4.6 h under all the tested temperatures. Thus, the initial curing time of the HMGC is chosen to be 5 h before demolding and high temperature curing.

### 3.4. Compressive Strength of HMGC

**Figure 5:**
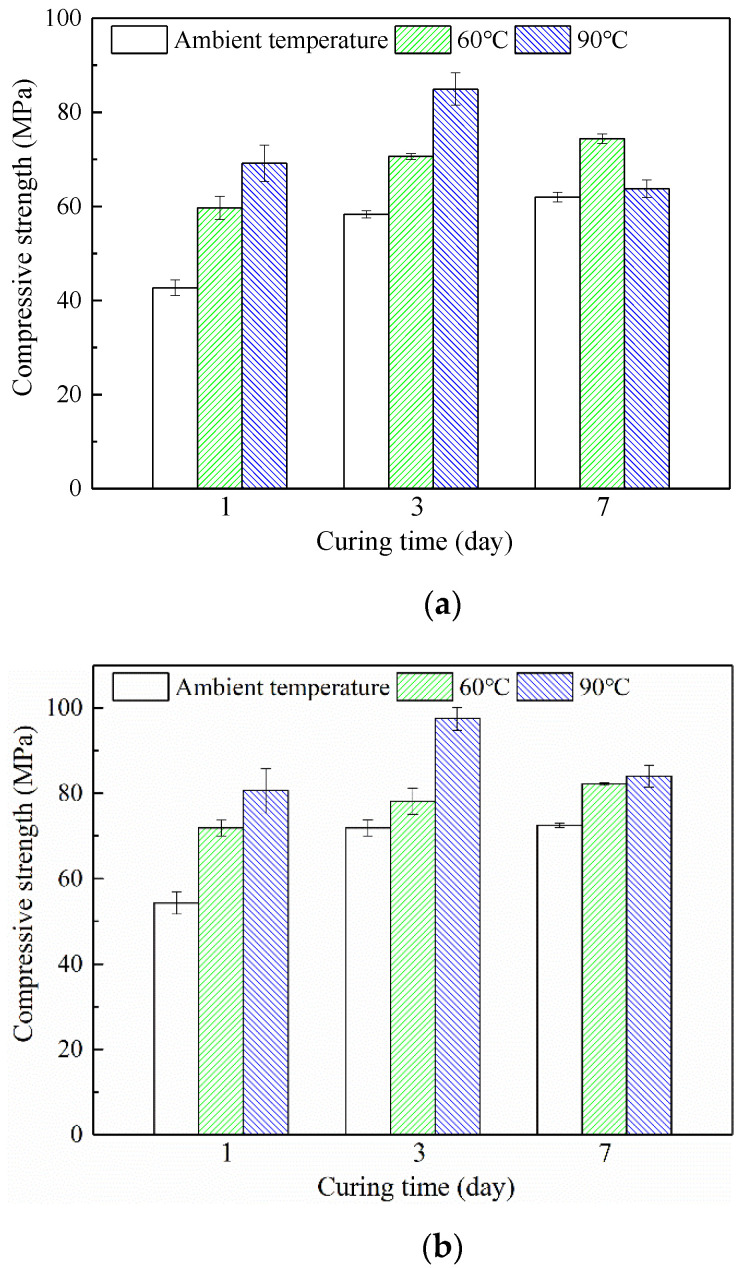
Compressive strength of HMGC under different curing conditions. (**a**) HMGC without steel fibers cured at different temperatures; (**b**) HMGC with steel fibers cured at different temperatures.

Figure 5 shows the change in compressive strength of the HMGC under different curing conditions. Different curing temperatures have different effects on the development of compressive strength of the HMGC. When cured at an ambient temperature, the compressive strength increases from 42.7 MPa to 62.0 MPa of the 7-day strength. At a temperature of 60 °C, the compressive strength increases from 59.7 MPa to 74.4 MPa. The same phenomenon has also been demonstrated in previous experiments, such as those conducted by Mo et al. [22] and Nath et al. [28]. They found that appropriate heat curing improves the early strength of geopolymers, but geopolymers cured at higher temperatures (e.g., 80 °C and 100 °C) inhibit the compactness and toughness of the structure due to excessive hardening rates. This is different from the findings of this paper; even under curing at 90 °C, the 1-day compressive strength of the geopolymer increased compared to 60 °C, which may be attributed to the maintenance of humidity and the mix ratio of materials. 

When cured at 90 °C, the 1-day compressive strength was 69.2 MPa and the 3-day compressive strength was 84.9 MPa. However, the 7-day compressive strength dropped to 63.8 MPa, which was lower than the 1-day strength. Similarly, the addition of steel fibers enhanced the compressive strength of the HMGC, and the compressive strengths after 1-day curing at ambient temperature, 60 °C and 90 °C reached 54.3 MPa, 71.9 MPa and 80.6 MPa, respectively. The compressive strength after curing at 90 °C for 3 days was 97.5 MPa, but the same result was obtained with the HMGC without fibers, and the compressive strength decreased after curing for 7 days. 

This result shows that increasing the temperature can promote the early strength of the HMGC (e.g., 60 and 90 °C, 1 and 3 days). Under ambient temperature, the dissolution rate of monomers and the polymerization rate of the aluminosilicate gel is low, resulting in the under-developed strength of the HMGC. The increase in temperature accelerates the dissolution and polymerization rate and improvement of the strength due to the thermodynamic effect, which is consistent with the research results of Bakharev et al. [29] and Muñiz-Villarreal et al. [30]. However, if the temperature is too high and the curing time is too long (e.g., 90 °C, 7 days), the compressive strength tends to decline. The reasons will be further analyzed with the help of microstructure characterizations and described in later sections.

### 3.5. Flexural Strength Analysis of HMGC

**Figure 6:**
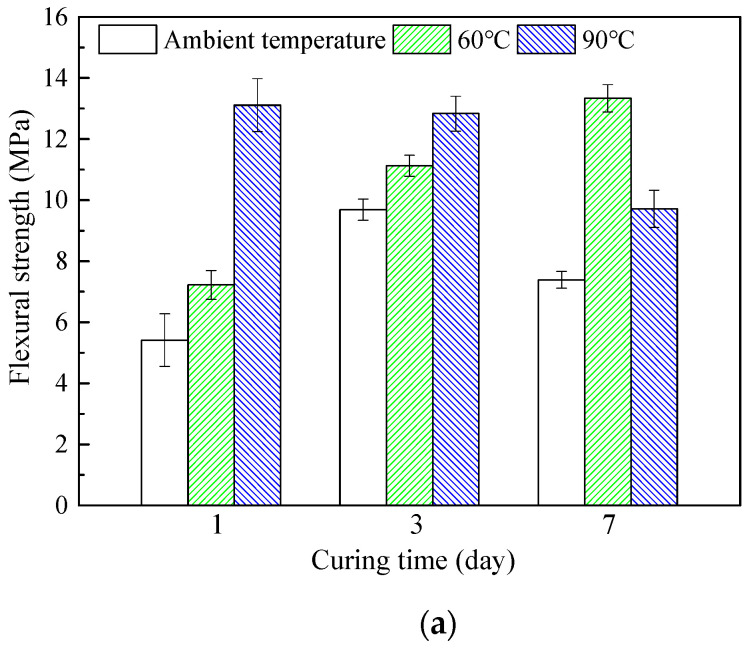

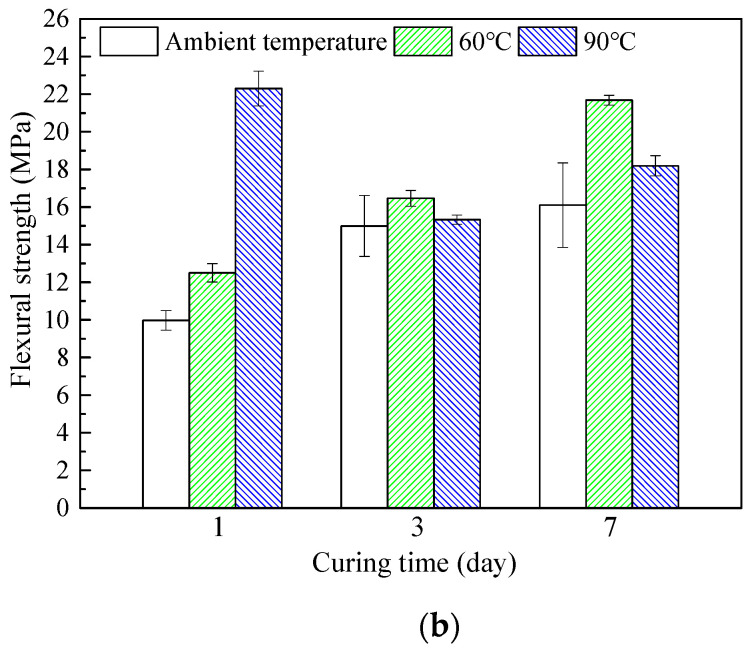
Flexural strength of HMGC under different curing conditions. (**a**) HMGC without steel fibers cured at different temperatures; (**b**) HMGC with steel fibers cured at different temperatures.

**Figure 7:**
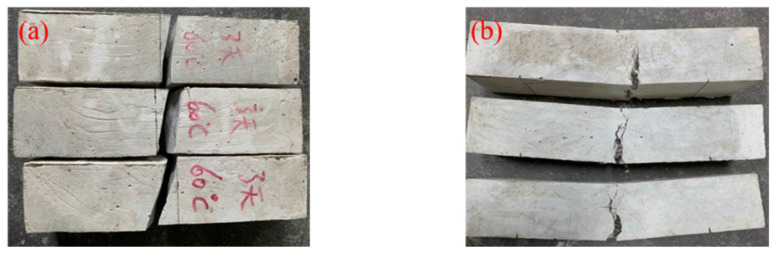
Morphology after flexural test of HMGC. (**a**) HMGC without steel fibers cured at 60 °C for three days; (**b**) HMGC with steel fibers.

Figure 6 shows the changing trend of the flexural strength of the HMGC at different curing temperatures. The addition of steel fibers enhances the flexural strength of the HMGC. After 1-day ambient temperature curing, the flexural strength of HMGC without steel fibers is only 5.4 MPa, while that of the HMGC with steel fibers is 9.97 MPa. Under a curing temperature of 60 °C, the flexural strength of 1 d without steel fibers is 7.2 MPa, and the 7 d flexural strength is 13.3 MPa. Under the same conditions, the 1-day flexural strength increases to 12.5 MPa and the 7-day flexural strength increases to 21.7 MPa by adding steel fibers. The HMGC without steel fibers cured at 90 °C for 1 day has the flexural strength of 13.1 MPa. Under the same conditions, the flexural strength of the HMGC with steel fibers increases to 22.3 MPa. Fracture failure occurs without fibers, but this does not occur after fibers are added (Figure 7). The same phenomenon has been demonstrated in previous research, such as that of Bhutta et al. [31] and Asrani et al. [32]. Therefore, these results confirm that adding steel fibers is useful for future applications of the HMGC. 

On the other hand, it was found that the flexural strength of a HMGC cured under 90 °C had a significant decrease in flexural strength when the curing process was prolonged (22.3 MPa for 1 d and 18.19 MPa for 7 d). This harmful effect largely eliminates the beneficial effect of fiber addition. Thus, for a HMGC with steel fibers, the curing temperature should be strictly controlled. The underlying mechanisms will be analyzed in later sections.

### 3.6. Micromorphology-SEM Analysis of HMGC

**Figure 8:**
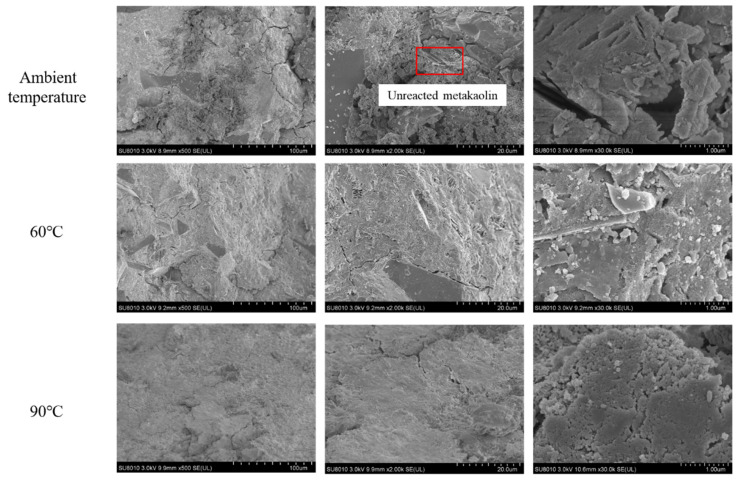
SEM images of HMGC under different curing temperatures at 7 d.

Figure 8 shows the microscopic morphology of the HMGC under different curing temperatures. Under ambient temperature, quartz powder, unreacted metakaolin and geopolymer gel can be observed in the sample. The unreacted metakaolin was found to be widely distributed in the gel matrix. In comparison, when cured at 60 °C, the amount of unreacted metakaolin was largely reduced. The overall structure was denser and fewer pores were shown in the gel matrix. Thermal curing greatly promotes the dissolution of metakaolin and generates more gels to bind the quartz powder. This results in an increased mechanical strength of the HMGC under the curing condition of 60 °C. When the curing temperature reaches 90 °C, the amount of unreacted metakaolin is further reduced. However, more pores and micro-defects are shown in the gel phase. This change in pore structure is further examined in the following section. 

### 3.7. Pore Structure Analysis of HMGC

**Figure 9:**
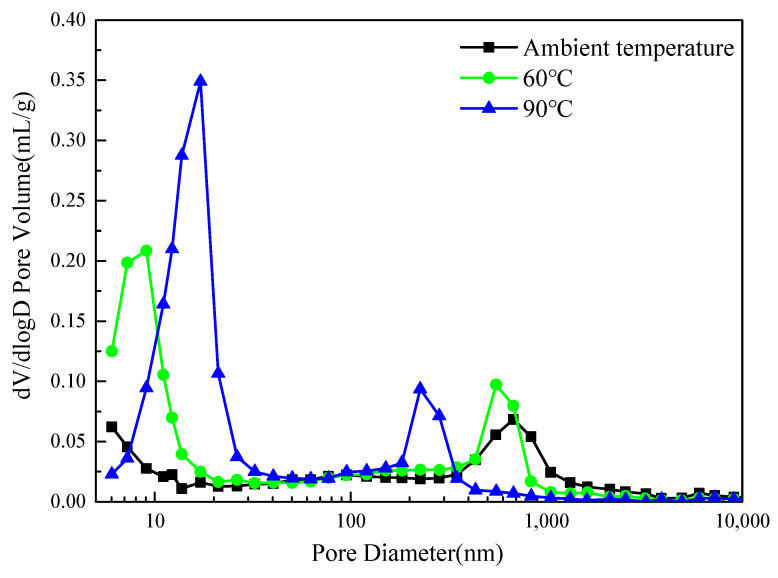
Pore size distribution of HMGC under different curing temperatures.

Table 5 shows the pore structure of the HMGC under different curing temperatures, obtained from MIP test. The porosity gradually increased from 13.6% to 25.6% with the increase in curing temperature. From the distribution curves (Figure 9), this increase in porosity is mainly due to the increase in small gel pores with a size of 6–30 nm. Moreover, as the curing temperature increases, the characteristic size of these pores (indicated by the location of the local curve peak) shifts to a larger size. This indicates that high-temperature curing coarsens the pores in the gel phase. Previous studies have shown that metakaolin-based geopolymers exhibit chemical shrinkage during the formation of silica-rich gels [33]. Therefore, an increased curing temperature and curing time would exacerbate the chemical shrinkage of the geopolymer gel and thus form more large gel pores and micro-defects (as shown in SEM morphologies). This would also lead to more interfacial defects between the gel phase (shrinkage) and quartz powder/steel fibers (non-deformed). Thus, the strength may be reduced as the high-temperature curing is prolonged (as shown in strength tests). 

On the other hand, Figure 9 also shows that the characteristic size of pores with a size of 200–1000 nm shift to a lower size with an increase in the curing temperature. This indicates that the gel fills more mesopores between the quartz powder, which results from the thermal promotion of the dissolution and diffusion of the monomers [34]. This also leads to a more integrated matrix of the HMGC. The combination of the gel pore coarsening effect and the mesopore filling effect leads to an optimized curing temperature of 60 °C.

### 3.8. Phase Analysis of HMGC

**Figure 10:**
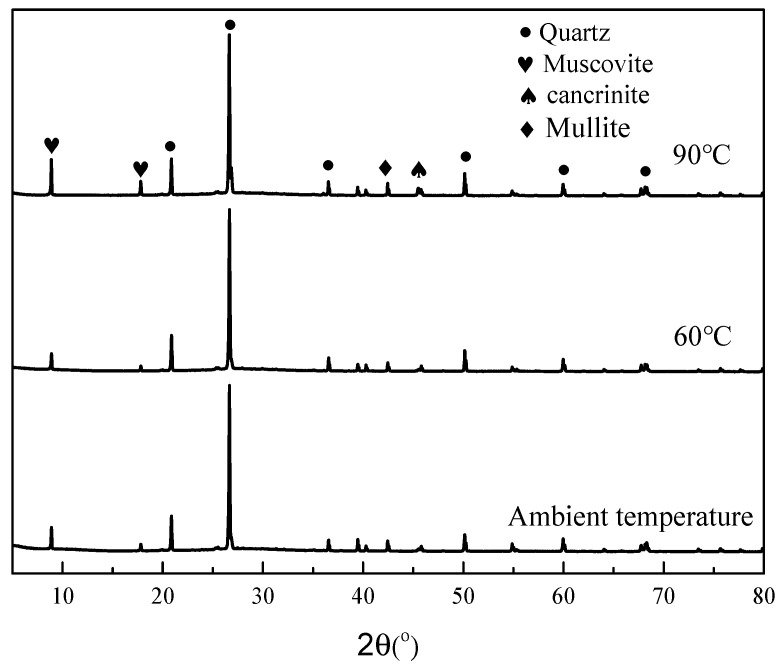
XRD curves of HMGCs under different curing temperatures.

Figure 10 shows the XRD patterns of the HMGC under different curing temperatures. Because the incorporation of fibers does not affect the final product, only the HMGC without steel fibers is analyzed. XRD patterns show that the main crystals of the HMGC under different curing temperatures are basically the same. The HMGC mainly contains quartz, muscovite, cancrinite and mullite, which are mainly present in their raw material powders. No new crystals were produced under high-temperature curing. The XRD results show that increasing the curing temperature does not have a great effect on the chemical structure of the HMGC. The strength variation of the HMGC is mainly attributed to the microstructure changes.

### 3.9. FTIR Results Analysis of HMGC

**Figure 11:**
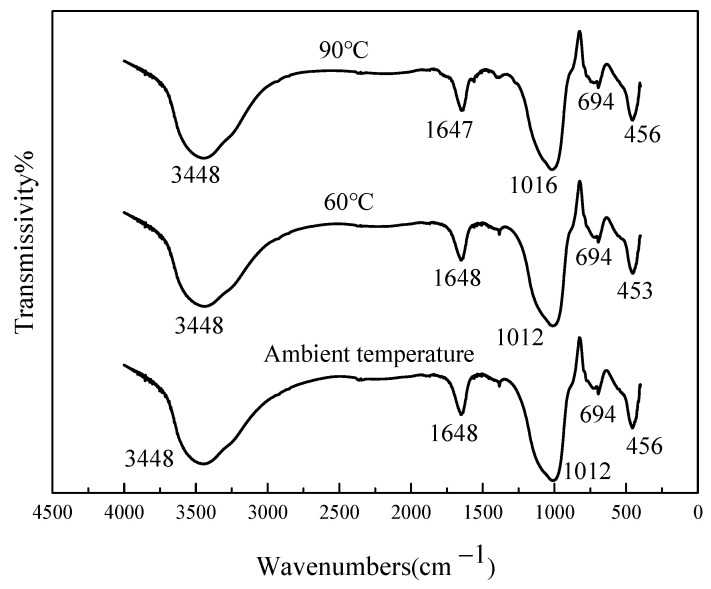
FTIR curves of HMGC under different curing temperatures.

Figure 11 shows the FTIR results of HMGCs cured at different temperatures. According to previous studies [3,35], it is believed that the absorption peaks of the HMGC at 3448 cm^−1^ are mainly the stretching vibration peaks of -OH. The absorption peaks at 1648 cm^−1^ and 1647 cm^−1^ are mainly stretching vibration peaks of H-O-H, and the existence of these two peaks is mainly due to the presence of H_2_O. Meanwhile, the absorption peaks at 1200–900 cm^−1^ are mainly the asymmetric stretching vibrations of Si-O-T (T = Al, Si) [36]; the absorption peaks at 1012 cm^−1^ and 1016 cm^−1^ are Si-O-Al and the asymmetric vibration of Si-O-Si corresponds to a shift in the wave number to a lower frequency, indicating that more aluminum-oxygen tetrahedra replace silicon-oxygen tetrahedra. The absorption peaks in the range of 720–650 cm^−1^ are thought to be due to the symmetrical vibrations of the tetrahedral groups (SiO_4_ and AlO_4_) of some zeolites [35]. The absorption peaks in the range of 450–460 cm^−1^ are mainly due to the symmetrical stretching vibration of Si-O-Si or Al-O-Si bonds [37,38].

Based on the previous FTIR spectra, it can be found that the HMGC cured at different temperatures showed absorption peaks at 3448 cm^−1^ and 1648 cm^−1^, mainly due to the stretching vibration of -OH and H-O-H. Under the action of alkali activator, the silicon-oxygen tetrahedron and aluminum-oxygen tetrahedron in metakaolin are released and recombined to form the geopolymer gel. The band at 694 cm^−1^ indicates the presence of a zeolite precursor (amorphous aluminosilicate network structure) in the sample, but it is practically impossible to use these peaks to distinguish the zeolite present. It can therefore be inferred that the existing amorphous matrix and crystalline zeolites have similar properties in all samples. Comparing FTIR at different temperatures, the absorption peak appeared at 1016 cm^−1^ at 90 °C, and the absorption peak appeared at 1012 cm^−1^ at ambient temperature and 60 °C. It was found that when the curing temperature was 60 °C, the wave number of the absorption peak which appeared near 1012 cm^−1^ was the smallest, so it can be inferred that the degree of geopolymerization of the HMGC cured at 60 °C is relatively high, which is consistent with the results for mechanical strength.

## 4. Conclusions

This paper mainly studies the optimized composition of HMGC with quartz powder and steel fibers, and the effect of curing temperature on this proposed new material. Combined with XRD, FTIR, SEM and MIP characterizations, the underlying mechanisms behind the effects of curing temperature are analyzed as well. The main conclusions drawn are as follows:

(1) The optimized quartz powder/metakaolin ratio is 1.5 and steel fibers content is 1.5 vol.%, according to the mechanical performance of the HMGC.
(2) The curing temperature has little effect on the crystalline product and chemical structures of the HMGC. The effect of curing temperature on strength is majorly due to microstructure changes.
(3) Heat curing at an appropriate temperature (such as 60 °C) can significantly speed up the process of geopolymerization and enhance the pore-filling effect of the gel. This leads to a high mechanical strength of the HMGC. However, long-term heat curing under too-high temperature (such as 90 °C, 7 days) would coarsen the pores and cause micro-defects in the gel phase of the HMGC due to chemical shrinkage, thus reducing the mechanical performance of the HMGC.
(4) The curing temperature should be carefully controlled to produce a HMGC with better performance in applications.

## Figures and Tables

**Table 1:** Chemical components of metakaolin.

Content	SiO_2_	Al_2_O_3_	TiO_2_	Fe_2_O_3_	Na_2_O	K_2_O	CaO	LOI
wt.%	57.46	39.81	1.79	0.43	0.27	0.21	0.04	0.34

**Table 2:** Physical properties of steel fibers.

Length (mm)	Diameter (mm)	Density (g/cm^3^)	Elastic Modulus (GPa)	Tensile Strength (MPa)
15	0.2	7.9	200	2850

**Table 3:** Composition of HMGC with different quartz powder contents (unit: g).

Number	Quartz Powder/Metakaolin Ratio	Water Glass	NaOH	Metakaolin	Quartz Powder
1	0	295.2	47.4	225.0	0
2	0.5	295.2	47.4	225.0	112.5
3	1.0	295.2	47.4	225.0	225.0
4	1.5	295.2	47.4	225.0	337.5
5	2.0	295.2	47.4	225.0	450.0

**Table 4:** Composition of HMGC with different fiber contents (unit: g).

Number	Fibers Volume Fraction	Water Glass	NaOH	Metakaolin	Quartz Powder	Steel Fibers
1	0	590.4	94.9	450.0	675.0	0
2	0.5%	590.4	94.9	450.0	675.0	30.3
3	1.0%	590.4	94.9	450.0	675.0	60.7
4	1.5%	590.4	94.9	450.0	675.0	91.0
5	2.0%	590.4	94.9	450.0	675.0	121.3

**Table 5:** Porosity of HMGC under different curing temperatures.

	Ambient Temperature	60 °C	90 °C
Porosity/%	13.6	20.3	25.6

## Data Availability

Not applicable.
